# Clinical phenotypes and multidomain sexual function in men with ejaculatory and orgasmic dysfunction: a cross-sectional analysis of the CLIMAX Registry

**DOI:** 10.1093/sexmed/qfag097

**Published:** 2026-10-23

**Authors:** Leona Hariharan, Sara Perelmuter, Regina Hill, Rachel S Rubin, Alan W Shindel

**Affiliations:** School of Medicine, University of California, San Francisco, San Francisco, CA 94122, United States; Weill Cornell Medicine, New York, NY 10065, United States; Sexual Medicine Research Team (SMRT), Washington, DC 20015, United States; Department of Urology, Georgetown University, Washington, DC 20015, United States; Department of Urology, University of California, San Francisco, San Francisco, CA 94122, United States

**Keywords:** delayed ejaculation, anorgasmia, erectile dysfunction, orgasm, ejaculation, sexual dysfunction, physiological

## Abstract

**Introduction:**

Delayed ejaculation (DE) and anorgasmia/anejaculation are frequently grouped together, yet whether they represent distinct clinical phenotypes or points along a shared spectrum remains unclear. Relationships between erectile function and other sexual function domains have been characterized primarily in men with erectile dysfunction rather than ejaculatory or orgasmic dysfunction. We sought to characterize clinical phenotypes and multidomain sexual function among men with ejaculatory or orgasmic dysfunction using baseline data from the CLIMAX Registry.

**Methods:**

We performed a cross-sectional secondary analysis of baseline data from 43 participants enrolled in the prospective, multi-institutional CLIMAX Registry. The phenotype analysis included isolated DE (*n* = 14) and isolated anorgasmia/anejaculation (*n* = 13), defined as selection of these diagnoses without another ejaculatory/orgasmic diagnosis. These groups were compared using clinical, psychosocial, and sexual function characteristics. A separate analysis of the broader registry cohort evaluated relationships between erectile function and other sexual function domains. Participants with complete International Index of Erectile Function erectile function domain (IIEF-EF) scores were stratified by erectile function severity (IIEF-EF ≥17 [none/mild erectile dysfunction] vs IIEF-EF ≤16 [moderate/severe erectile dysfunction]). Continuous variables were compared using Mann–Whitney U tests, categorical variables using Fisher exact tests, and erectile function and orgasmic function were evaluated using Pearson correlation.

**Results:**

The overall cohort included 43 participants (mean age, 56.4 ± 15.7 years). Participants with isolated anorgasmia/anejaculation were significantly older than those with isolated DE (67.6 ± 14.6 vs 53.3 ± 12.8 years; *P* = .004). Beck Depression Inventory scores were higher among participants with DE (14.3 ± 12.3 vs 4.8 ± 4.1; *P* = .013), whereas standardized sexual function domains did not differ significantly between phenotypes. Notably, 6 of 13 participants (46.2%) in the combined anorgasmia/anejaculation category reported ability to achieve orgasm. Erectile and orgasmic function were not significantly correlated (*r* = −0.03; *P* = .84; *n* = 37), and 7 of 22 participants (31.8%) with unassisted erections sufficient for penetration reported inability to achieve orgasm. Moderate/severe erectile dysfunction was associated with lower intercourse satisfaction (*P* = .017) but not orgasmic function.

**Conclusions:**

Diagnostic phenotype and erectile function alone incompletely characterized sexual function in this exploratory cohort. These findings support multidimensional phenotyping of uncommon ejaculatory and orgasmic disorders and highlight the value of prospective registries for clinical characterization.

## Introduction

Ejaculatory dysfunction beyond premature ejaculation remains an understudied area despite its substantial impact on quality of life.^1^^,^^2^ Delayed ejaculation (DE) and anorgasmia are relatively uncommon, with prevalence estimates generally below 5%, but estimates vary due to inconsistent definitions and limited epidemiologic investigation.^3^ The AUA/SMSNA guideline notes that anorgasmia may represent an extreme variant of DE, whereas the ICD-11 classifies both conditions under a single diagnosis of male DE.^4^ Relatively little is known about whether these presentations represent distinct clinical phenotypes or points along a shared clinical spectrum. Existing literature often combined DE and anorgasmia into heterogeneous cohorts, limiting understanding of these individual conditions.^3^^,^^5^ Improved phenotypic characterization may facilitate more individualized management.^1^^,^^2^

Although orgasm and ejaculation typically occur together, they are physiologically distinct processes involving separate central and peripheral mechanisms.^6^ Erectile function, orgasmic function, sexual desire, and sexual satisfaction represent related but distinct domains of male sexual function.^7^^,^^8^ The International Index of Erectile Function (IIEF) provides a standardized multidomain assessment of these constructs, yet few studies have characterized these domains specifically among men with ejaculatory dysfunction.^9^ Relationships among these domains have been evaluated primarily in men with erectile dysfunction rather than disorders of ejaculation.^6^

The CLIMAX Registry was established to prospectively characterize men with ejaculatory and orgasmic dysfunction using standardized clinical data and validated patient-reported outcome measures. Using baseline registry data, we sought to characterize how diagnostic phenotype and erectile function relate to multidomain sexual function. We asked two complementary questions: do clinical, psychosocial, contextual, and sexual function characteristics differ between participants with isolated DE and isolated anorgasmia/anejaculation, and is erectile function associated with other sexual function domains, particularly orgasmic function, across the broader registry cohort?

## Methods

### Study design and participants

This cross-sectional secondary analysis used prospectively collected CLIMAX Registry baseline data from June 2024 through April 2026. Eligible participants were US men aged ≥18 years beginning treatment for an ejaculatory or orgasmic disorder and were recruited by urologists in academic and private-practice settings. Participants independently completed an online baseline questionnaire; partners could separately complete a partner questionnaire.

At baseline, participants reported their current diagnosis by selecting one or more predefined registry categories: premature ejaculation, DE, anorgasmia/anejaculation, or dysorgasmia/dysejaculation. For the phenotype analysis, participants selecting DE without another ejaculatory/orgasmic diagnosis were classified as isolated DE (*n* = 14), whereas those selecting anorgasmia/anejaculation without another diagnosis were classified as isolated anorgasmia/anejaculation (*n* = 13). Anorgasmia/anejaculation and dysorgasmia/dysejaculation were each presented as combined response categories in the source questionnaire. Participants selecting multiple or other diagnoses were excluded from this comparison.

For the broader erectile function analysis, all participants with complete IIEF erectile function domain (IIEF-EF) scores were included regardless of ejaculatory/orgasmic diagnosis (*n* = 39) and categorized as none/mild erectile dysfunction (IIEF-EF ≥ 17, *n* = 30) or moderate/severe erectile dysfunction (IIEF-EF ≤ 16, *n* = 9), consistent with established classifications.^10^ A separate correlation analysis included participants with complete paired IIEF-EF and orgasmic function domain scores (*n* = 37).

The CLIMAX Registry was approved by WCG Institutional Review Board (protocol #20241827) and conducted in accordance with the Declaration of Helsinki. All participants provided informed consent before enrollment.

### Clinical measures

Baseline measures included demographic and clinical characteristics, sexual function and activity, symptom severity, and relationship impact. Depressive symptoms were assessed using the 21-item Beck Depression Inventory (BDI; range: 0-63).

Sexual function was assessed using the 15-item IIEF. Domain scores were calculated as erectile function (items 1-5 and 15), orgasmic function (items 9-10), sexual desire (items 11-12), intercourse satisfaction (items 6-8), and overall satisfaction (items 13-14).^9^ In the primary analysis, responses coded as 0 on applicable items, indicating no sexual activity, intercourse, or attempt, were treated as missing, and domain scores were calculated only when all component items were available.

Global symptom severity was assessed using the Patient Global Impression of Severity (PGI-S). Ability to achieve orgasm and relationship impact were recorded as dichotomous patient-reported outcomes. Unassisted erection hardness was assessed on a 0-4 scale, with scores ≥3 indicating an erection sufficient for penetration.

Optional free-text responses were reviewed descriptively and categorized into clinical and contextual dimensions not fully captured by structured questionnaire items. Participants could contribute to multiple categories; unreported experiences were not assumed to be absent. No inferential comparisons were performed. Baseline partner questionnaires were linked to participant records and included separate assessments of perceived participant bother and the partner’s own bother. Partner-reported outcomes were summarized descriptively.

### Statistical analysis

Continuous variables are presented as mean (SD), with median (IQR) additionally reported for sexual function and BDI outcomes; categorical variables are presented as number (percentage). Group comparisons were performed using Mann–Whitney U tests for continuous variables and two-sided Fisher exact tests for categorical variables.

Associations between erectile function and orgasmic function were evaluated using Pearson correlation among participants with paired complete domain scores. As a complementary descriptive analysis, orgasm ability was characterized among participants with an unassisted erection hardness score ≥3.

Analyses were conducted using complete-case data. To assess the impact of IIEF responses scored 0, sensitivity analyses repeated IIEF domain comparisons and the erectile–orgasmic function correlation with 0 responses retained. Separate exploratory age-adjusted linear regression models were fitted for each IIEF domain, with phenotype and age as predictors.

Given the exploratory nature of the analyses, modest sample size, and multiple comparisons, no correction for multiple comparisons was applied. Two-sided *P* values <.05 were considered statistically significant. Statistical analyses were performed using Stata version 18 (StataCorp LLC).

## Results

### Phenotypic characterization of isolated DE and anorgasmia/anejaculation

The baseline registry cohort included 43 participants (mean age: 56.4 ± 15.7 years; Table S1). Among participants with isolated phenotypes, those with anorgasmia/anejaculation were significantly older than those with DE (67.6 ± 14.6 vs 53.3 ± 12.8 years, *P* = .004). Beck Depression Inventory scores were also higher among participants with DE (14.3 ± 12.3 vs 4.8 ± 4.1, *P* = .013), whereas relationship impact and other measured clinical characteristics did not differ significantly between groups (Table 1).

**Table 1:** Clinical, sexual function, and contextual characteristics of isolated delayed ejaculation and isolated anorgasmia/anejaculation.

**Panel A. Clinical, psychosocial, and sexual function characteristics**					
**Characteristic**	**Delayed ejaculation (*n* = 14)**	**Anorgasmia/anejaculation (*n* = 13)**		***P* value**	
**Clinical characteristics**					
Age, years, mean (SD)	53.3 (12.8)	67.6 (14.6)		**.004**	
Diabetes, *n* (%)	3 (21.4)	0 (0.0)		.222	
Hypogonadism, *n* (%)	11 (78.6)	6 (46.2)		.120	
Peripheral neuropathy, *n* (%)	1 (7.1)	3 (23.1)		.326	
Psychiatric history, *n* (%)	1 (7.1)	0 (0.0)		1.000	
Prior prostate/pelvic surgery, *n* (%)	2 (14.3)	2 (15.4)		1.000	
Antidepressant use, *n* (%)	5 (35.7)	1 (7.7)		.165	
Testosterone therapy, *n* (%)	11 (78.6)	5 (38.5)		.054	
PDE5 inhibitor use, *n* (%)	11 (78.6)	7 (53.8)		.236	
**Psychosocial characteristics**					
BDI score, mean (SD); median (IQR)	14.3 (12.3); 11.0 (6.0-14.0)	4.8 (4.1); 4.5 (1.5-7.2)		**.013**	
Sexual function negatively impacts relationship, *n* (%)	10 (71.4)	7 (53.8)		.345	
**Sexual function**					
Erectile function (IIEF-EF)	20.8 (7.0); 23.0 (15.0-26.0)	24.3 (7.1); 26.0 (20.0-30.0)		.151	
Orgasmic function	5.2 (2.6); 4.0 (4.0-8.0)	4.0 (1.1); 4.0 (4.0-4.0)		.314	
Sexual desire	6.9 (2.7); 8.0 (5.0-9.0)	7.4 (2.9); 8.0 (6.5-10.0)		.563	
Intercourse satisfaction	8.6 (2.9); 8.0 (6.0-11.0)	8.2 (2.5); 7.5 (6.2-9.8)		.755	
Overall satisfaction	5.6 (2.2); 5.0 (4.0-8.0)	4.8 (2.4); 4.5 (2.8-6.2)		.395	
PGI-S	3.4 (0.8); 4.0 (3.0-4.0)	3.8 (0.4); 4.0 (4.0-4.0)		.102	
Able to achieve orgasm, *n* (%)	11 (78.6)	6 (46.2)		.120	
**Panel B. Exploratory contextual characteristics from optional free-text responses**					
**Explicitly reported contextual dimension**	**Isolated delayed ejaculation (*n* = 7)**	**Isolated anorgasmia/anejaculation (*n* = 5)**	**Total (*n* = 12)**	**Illustrative participant response**	
Orgasm/ejaculation varied by sexual context or mode of stimulation	4	3	**7**	“The only way I’m able to orgasm is through masturbation after sex.”	
Preserved or treatment-assisted erectile function despite persistent orgasmic/ejaculatory difficulty	3	2	**5**	“I don’t have an issue achieving and maintaining an erection… I can’t have an orgasm without manually stimulating myself.”	
Partner or relationship factors influenced sexual activity or function	2	3	**5**	“There is usually no orgasm as my partner will eventually express her desire for the marathon session to end.”	
Structured questionnaire did not fully capture sexual experience or context	2	2	**4**	“Also, I assume ‘sexual stimulation’ includes masturbation?”	

IIEF domain, PGI-S, and BDI values are mean (SD); median (IQR) unless otherwise indicated. BDI data were available for 13 participants with isolated delayed ejaculation and 12 with isolated anorgasmia/anejaculation. IIEF complete-case sample sizes for isolated delayed ejaculation/anorgasmia-anejaculation were 13/11 for erectile function, 13/10 for orgasmic function, 13/12 for sexual desire, 13/10 for intercourse satisfaction, 13/12 for overall satisfaction, and 14/13 for PGI-S. Mann–Whitney U tests were used for continuous variables and two-sided Fisher exact tests for categorical variables.

Optional free-text responses containing substantive contextual information were available from 12 participants (7 with isolated delayed ejaculation and 5 with isolated anorgasmia/anejaculation). Participants could contribute to more than one category. Because responses were optional, counts reflect explicitly reported experiences among available narratives rather than prevalence estimates. Illustrative quotations are reproduced verbatim from deidentified participant responses; ellipses indicate omitted text.

Abbreviations: BDI, Beck Depression Inventory; IIEF, International Index of Erectile Function; IIEF-EF, IIEF Erectile Function domain; IQR, interquartile range; PDE5, phosphodiesterase type 5; PGI-S, Patient Global Impression of Severity.

Mean IIEF domain scores, PGI-S scores, and proportion reporting ability to achieve orgasm did not differ significantly between phenotypes (all *P* > .05; Table 1). Notably, 6 of 13 participants (46.2%) in the combined anorgasmia/anejaculation category reported being able to achieve orgasm. In sensitivity analyses retaining IIEF responses scored 0, between-group differences remained nonsignificant across all IIEF domains (Table S2); no IIEF domain differed significantly in exploratory age-adjusted analyses.

Substantive free-text responses were available from 12 participants. Responses described variation in orgasmic/ejaculatory function by sexual context or mode of stimulation (*n* = 7) and preserved or treatment-assisted erectile function despite persistent orgasmic/ejaculatory difficulty (*n* = 5) (Table 1). Illustrative responses included, “The only way I’m able to orgasm is through masturbation after sex” and “I don’t have an issue achieving and maintaining an erection… I can’t have an orgasm without manually stimulating myself.”

Among 5 partners of participants with isolated DE, 4 perceived the participant as very bothered, whereas only 1 reported being very bothered themselves.

### Erectile function in relation to orgasmic and other sexual function domains

When participants were stratified by erectile function severity, moderate/severe erectile dysfunction was associated with lower intercourse satisfaction (*P* = .017), with trends toward lower sexual desire (*P* = .070) and overall satisfaction (*P* = .051); orgasmic function and PGI-S did not differ significantly (Table 2).

**Table 2:** Erectile and orgasmic function in the broader CLIMAX Registry cohort.

**Panel A. Sexual function according to erectile dysfunction severity**			
**Outcome**	**None/mild erectile dysfunction (IIEF-EF ≥ 17; *n* = 30)**	**Moderate/severe erectile dysfunction (IIEF-EF ≤ 16; *n* = 9)**	***P* value**
Orgasmic function	5.9 (2.7); 5.0 (4.0-8.0)	5.4 (3.1); 4.0 (3.8-7.0)	.559
Sexual desire	7.9 (2.2); 9.0 (7.0-9.8)	6.1 (2.8); 6.0 (4.0-8.0)	.070
Intercourse satisfaction	8.9 (2.6); 8.0 (7.0-11.0)	6.2 (2.3); 6.0 (4.8-8.0)	.017
Overall satisfaction	5.4 (2.3); 5.0 (4.0-7.8)	3.7 (1.4); 4.0 (2.0-4.0)	.051
PGI-S	3.5 (0.6); 4.0 (3.0-4.0)	3.7 (0.5); 4.0 (3.0-4.0)	.423
**Panel B. Complementary measures of erectile–orgasmic function**			
**Analysis**		**Result**	
Correlation between IIEF erectile and orgasmic function domains		*n* = 37; Pearson *r* = −0.03; *P* = .841	
Able to achieve orgasm among participants with unassisted erection hardness score ≥3		15/22 (68.2%)	
Unable to achieve orgasm despite unassisted erection hardness score ≥3		7/22 (31.8%)	

Values are mean (SD); median (IQR) unless otherwise indicated. Mann–Whitney U tests were used for continuous-variable comparisons. Complete-case sample sizes for the none/mild and moderate/severe erectile dysfunction groups, respectively, were 29/8 for orgasmic function, 30/9 for sexual desire, 29/8 for intercourse satisfaction, 30/9 for overall satisfaction, and 30/9 for PGI-S. The erectile function–orgasmic function correlation included participants with paired complete domain scores (*n* = 37). Erection hardness score ≥3 indicates an unassisted erection sufficient for penetration.

Abbreviations: ED, erectile dysfunction; IIEF, International Index of Erectile Function; IIEF-EF, IIEF Erectile Function domain; PGI-S, Patient Global Impression of Severity.

Among participants with paired complete data, erectile function was not significantly correlated with orgasmic function (Pearson *r* = −0.03, *P* = .84; *n* = 37). Among 22 participants reporting an unassisted erection hardness score ≥3, 7 (31.8%) nevertheless reported inability to achieve orgasm (Table 2).

When IIEF responses scored 0 were retained, differences in sexual desire (*P* = .014), intercourse satisfaction (*P* = .003), and overall satisfaction (*P* = .042) were statistically significant, whereas orgasmic function remained nonsignificant (*P* = .116). The erectile function–orgasmic function correlation also remained nonsignificant (*r* = .206, *P* = .196; *n* = 41; Table S2).

## Discussion

This exploratory analysis highlights the complexity of characterizing uncommon ejaculatory and orgasmic disorders and the value of prospective registry data. Isolated DE and anorgasmia/anejaculation differed in age and depressive symptom burden but not standardized sexual function domains. Nearly half of participants selecting the combined anorgasmia/anejaculation category reported ability to achieve orgasm, and patient narratives identified contextual features not fully reflected by structured measures. Together, these findings suggest that conventional diagnostic labels and individual sexual function domains capture complementary, but incomplete, aspects of these presentations.

Few studies have evaluated DE and anorgasmia/anejaculation as separate phenotypes using standardized patient-reported outcomes.^1^^,^^2^ Importantly, anorgasmia and anejaculation were combined despite representing distinct components of sexual climax; 6 of 13 participants in this category reported ability to achieve orgasm. Free-text responses similarly described context-dependent symptoms and preserved erectile function despite orgasmic or ejaculatory difficulty. Higher BDI scores among participants with DE warrant cautious interpretation but suggest that psychosocial characteristics may provide additional information. Limited partner data and patient narratives further support considering individual and relational context. Together, these findings highlight the need for more granular phenotyping that distinguishes orgasm from ejaculation and captures symptom context.

Our findings reinforce that erectile function and orgasmic function represent distinct domains of sexual health.^8^ Previous studies suggest that ejaculatory and orgasmic dysfunction may occur across erectile function severity and are not uniformly linked.^5^^,^^7^ Consistent with this literature, erectile dysfunction severity was associated with satisfaction but not orgasmic function, and erectile and orgasmic function were not significantly correlated. Moreover, nearly one-third of participants with unassisted erections sufficient for penetration nevertheless reported inability to achieve orgasm.

These findings support multidimensional assessment in men with ejaculatory and orgasmic dysfunction. Erectile function, orgasmic function, desire, satisfaction, and relevant psychosocial or relational context may provide complementary information beyond diagnosis or erectile dysfunction severity. Prospective, multi-institutional registries, such as CLIMAX, can build granular cohorts of uncommon conditions that are difficult to characterize in single-center studies. Continued enrollment and longitudinal follow-up may help to distinguish phenotypes from overlapping or evolving presentations and to identify subgroups for mechanistic and therapeutic investigation.

Limitations include modest sample size, cross-sectional design, and reduced sample sizes for some complete-case analyses. Diagnostic categories were patient-reported, and participants could select multiple diagnoses; restricting the phenotype comparison to isolated categories may limit generalizability to patients with overlapping symptoms. The questionnaire did not systematically distinguish lifelong vs acquired or generalized vs situational symptoms. Age differences and other unmeasured characteristics may contribute to residual confounding. Free-text and partner-reported findings were exploratory and based on limited responses. The predominantly White and heterosexual cohort also limits generalizability.

## Conclusion

These findings support multidimensional phenotyping beyond diagnostic labels or individual sexual function domains and highlight the value of prospective registries for characterizing uncommon ejaculatory and orgasmic disorders.

## Supplementary Material

Supplemental_Tables_Climax_qfag097
