# Supplementary material for: Clinical phenotypes and multidomain sexual function in men with ejaculatory and orgasmic dysfunction: a cross-sectional analysis of the CLIMAX Registry
Source: Sex Med. 2026 Oct 23;14(6):qfag097. doi: 10.1093/sexmed/qfag097 (PMC13693590; doi:10.1093/sexmed/qfag097)
Supplement: Supplementary file 1 [file supplemental_tables_climax_qfag097.docx]

**Supplementary Table 1. Baseline Characteristics of the Overall CLIMAX Registry Cohort**

| **Characteristic** | **Overall (N = 43)** |
| --- | --- |
| Age, years, mean (SD) | 56.4 (15.7) |
| **Diagnosis, n (%)** |  |
| Delayed ejaculation | 14 (32.6) |
| Anorgasmia/Anejaculation | 13 (30.2) |
| Delayed ejaculation + Anorgasmia/Anejaculation | 6 (14.0) |
| Premature ejaculation | 8 (18.6) |
| Premature ejaculation + Delayed ejaculation | 1 (2.3) |
| Premature ejaculation + Dysorgasmia/Dysejaculation | 1 (2.3) |
| **Sexual orientation, n/N (%)†** |  |
| Heterosexual/Straight | 34/42 (81.0) |
| Bisexual | 6/42 (14.3) |
| Gay | 2/42 (4.8) |
| **Race, n/N (%)†** |  |
| White/European/Caucasian | 37/42 (88.1) |
| Other | 3/42 (7.1) |
| South Asian | 1/42 (2.4) |
| Native American/American Indian | 1/42 (2.4) |
| **Ethnicity, n/N (%)†** |  |
| Not Latino/Hispanic/Chicano | 39/41 (95.1) |
| Latino/Hispanic/Chicano | 2/41 (4.9) |
| **Clinical characteristics, n (%)** |  |
| Diabetes | 4 (9.3) |
| Hypogonadism | 25 (58.1) |
| Peripheral neuropathy | 5 (11.6) |
| Psychiatric history | 1 (2.3) |
| Prior prostate/pelvic surgery | 6 (14.0) |
| Antidepressant use | 10 (23.3) |
| Testosterone therapy | 22 (51.2) |
| PDE5 inhibitor use | 28 (65.1) |

Abbreviations: PDE5, phosphodiesterase type 5; SD, standard deviation.

† Percentages were calculated using available-case denominators. Testosterone therapy and PDE5 inhibitor use were obtained from questionnaire responses; antidepressant use was identified from medication text.

**Supplementary Table 2. Sensitivity Analyses Retaining IIEF Responses Scored 0**

**Panel A. Isolated Delayed Ejaculation vs Isolated Anorgasmia/Anejaculation**

| **IIEF Domain** | **Isolated Delayed Ejaculation (n = 13)** | **Isolated Anorgasmia/Anejaculation (n = 12)** | **P value** |
| --- | --- | --- | --- |
| Erectile Function | 20.8 (7.0); 23.0 (15.0–26.0) | 22.3 (9.6); 26.0 (19.0–30.0) | 0.323 |
| Orgasmic Function | 5.2 (2.6); 4.0 (4.0–8.0) | 3.5 (1.6); 4.0 (2.8–4.0) | 0.121 |
| Sexual Desire | 6.9 (2.7); 8.0 (5.0–9.0) | 7.4 (2.9); 8.0 (6.5–10.0) | 0.563 |
| Intercourse Satisfaction | 8.6 (2.9); 8.0 (6.0–11.0) | 6.8 (3.9); 7.0 (5.8–9.2) | 0.325 |
| Overall Satisfaction | 5.6 (2.2); 5.0 (4.0–8.0) | 4.8 (2.4); 4.5 (2.8–6.2) | 0.395 |

**Panel B. Erectile Dysfunction Severity**

| **IIEF Domain** | **None/Mild Erectile Dysfunction (n = 30)** | **Moderate/Severe Erectile Dysfunction (n = 11)** | **P value** |
| --- | --- | --- | --- |
| Orgasmic Function | 5.8 (2.8); 5.0 (4.0–8.0) | 4.3 (3.3); 4.0 (2.5–5.0) | 0.116 |
| Sexual Desire | 7.9 (2.2); 9.0 (7.0–9.8) | 5.4 (3.0); 5.0 (2.5–8.0) | 0.014 |
| Intercourse Satisfaction | 8.6 (3.0); 8.0 (7.0–11.0) | 5.0 (3.2); 5.0 (3.5–7.0) | 0.003 |
| Overall Satisfaction | 5.4 (2.3); 5.0 (4.0–7.8) | 3.7 (1.6); 4.0 (2.0–4.5) | 0.042 |

**Panel C. Erectile Function–Orgasmic Function Correlation**

| **Paired n** | **Pearson r** | **P value** |
| --- | --- | --- |
| 41 | 0.206 | 0.196 |

Note: Values are mean (SD); median (IQR). IIEF responses scored 0 were retained in domain scoring for this sensitivity analysis; all component items were otherwise required to calculate domain scores. Responses scored 0 indicated no sexual activity, no intercourse, or no attempt, as applicable. Mann–Whitney U tests were used for group comparisons; the erectile function–orgasmic function association was evaluated using Pearson correlation.
